# Source-Specific Air Pollution and Loss of Independence in Older Adults Across the US

**DOI:** 10.1001/jamanetworkopen.2024.18460

**Published:** 2024-06-28

**Authors:** Boya Zhang, Carlos F. Mendes de Leon, Kenneth M. Langa, Jennifer Weuve, Adam Szpiro, Jessica Faul, Jennifer D’Souza, Joel D. Kaufman, Richard A. Hirth, Lynda D. Lisabeth, Jiaqi Gao, Sara D. Adar

**Affiliations:** 1 Department of Epidemiology, University of Michigan School of Public Health, Ann Arbor; 2 Department of Oncology, Georgetown University, Washington, District of Columbia; 3 Institute for Social Research, University of Michigan, Ann Arbor; 4 University of Michigan Medical School, Ann Arbor; 5 Institute for Healthcare Policy and Innovation, University of Michigan, Ann Arbor; 6 Veterans Affairs Center for Clinical Management Research, Ann Arbor, Michigan; 7 Department of Epidemiology, Boston University School of Public Health, Boston, Massachusetts; 8 Department of Biostatistics, University of Washington, Seattle; 9 Department of Epidemiology, University of Washington, Seattle; 10 Department of Environmental and Occupational Health Sciences, University of Washington, Seattle; 11 Department of Medicine, University of Washington, Seattle; 12 Department of Health Management and Policy, University of Michigan School of Public Health, Ann Arbor; 13 Department of Internal Medicine, University of Michigan, Ann Arbor

## Abstract

**Importance:**

Air pollution is a recognized risk factor associated with chronic diseases, including respiratory and cardiovascular conditions, which can lead to physical and cognitive impairments in later life. Although these losses of function, individually or in combination, reduce individuals’ likelihood of living independently, little is known about the association of air pollution with this critical outcome.

**Objective:**

To investigate associations between air pollution and loss of independence in later life.

**Design, Setting, and Participants:**

This cohort study was conducted as part of the Environmental Predictors Of Cognitive Health and Aging study and used 1998 to 2016 data from the Health and Retirement Study. Participants included respondents from this nationally representative, population-based cohort who were older than 50 years and had not previously reported a loss of independence. Analyses were performed from August 31 to October 15, 2023.

**Exposures:**

Mean 10-year pollutant concentrations (particulate matter less than 2.5 μm in diameter [PM_2.5]_ or ranging from 2.5 μm to 10 μm in diameter [PM_10-2.5_], nitrogen dioxide [NO_2_], and ozone [O_3_]) were estimated at respondent addresses using spatiotemporal models along with PM_2.5_ levels from 9 emission sources.

**Main Outcomes and Measures:**

Loss of independence was defined as newly receiving care for at least 1 activity of daily living or instrumental activity of daily living due to health and memory problems or moving to a nursing home. Associations were estimated with generalized estimating equation regression adjusting for potential confounders.

**Results:**

Among 25 314 respondents older than 50 years (mean [SD] baseline age, 61.1 [9.4] years; 11 208 male [44.3%]), 9985 individuals (39.4%) experienced lost independence during a mean (SD) follow-up of 10.2 (5.5) years. Higher exposure levels of mean concentration were associated with increased risks of lost independence for total PM_2.5_ levels (risk ratio [RR] per 1-IQR of 10-year mean, 1.05; 95% CI, 1.01-1.10), PM_2.5_ levels from road traffic (RR per 1-IQR of 10-year mean, 1.09; 95% CI, 1.03-1.16) and nonroad traffic (RR per 1-IQR of 10-year mean, 1.13; 95% CI, 1.03-1.24), and NO_2_ levels (RR per 1-IQR of 10-year mean, 1.05; 95% CI, 1.01-1.08). Compared with other sources, traffic-generated pollutants were most consistently and robustly associated with loss of independence; only road traffic-related PM_2.5_ levels remained associated with increased risk after adjustment for PM_2.5_ from other sources (RR per 1-IQR increase in 10-year mean concentration, 1.10; 95% CI, 1.00-1.21). Other pollutant-outcome associations were null, except for O_3_ levels, which were associated with lower risks of lost independence (RR per 1-IQR increase in 10-year mean concentration, 0.94; 95% CI, 0.92-0.97).

**Conclusions and Relevance:**

This study found that long-term exposure to air pollution was associated with the need for help for lost independence in later life, with especially large and consistent increases in risk for pollution generated by traffic-related sources. These findings suggest that controlling air pollution could be associated with diversion or delay of the need for care and prolonged ability to live independently.

## Key Points

**Question:**

Are long-term exposures to air pollution from different emission sources associated with loss of independence in later life?

**Findings:**

In this cohort study of 25 314 adults older than 50 years, higher residential levels of traffic-related air pollutants, including particulate matter less than 2.5 μm in diameter (PM_2.5_) from road traffic, PM_2.5_ from nonroad traffic, and nitrogen dioxide, were associated with greater risks of lost independence.

**Meaning:**

These findings suggest that traffic-related air pollution may be associated with the likelihood of needing help for lost independence among older adults and that interventions to reduce pollution exposure may be associated with a prolonged ability to live independently.

## Teaser

This cohort study investigates the association of long-term exposure to air pollution from various emission sources with loss of independence among older adults.

## Introduction

Functional decline is common in aging populations, arising from subclinical pathologies and chronic diseases across multiple organs.^1,2,3^ As functional limitations due to health or memory problems increase in severity, affected individuals can experience substantial difficulties in their ability to live independently without help. Loss in independence is important given that it often results in large emotional and economic burdens for older adults, their families, and society.^4,5,6^ Nearly 30% of Medicare enrollees in the US receive help due to health problems with mobility, routine household activities, or basic self-care activities.^7^ Given the large and growing older adult population, interventions to prevent or delay the need for help due to lost independence are becoming increasingly important.

Air pollutants have well-established relationships with chronic health conditions, including cardiovascular diseases (CVDs), respiratory diseases, and diabetes, as well as the pathophysiological processes that give rise to these conditions.^8,9,10,11^ Chronic disease often leads to impairments in physical and cognitive function through mechanisms such as increased pain, reduced lung function, poor circulation, and disturbed sleep that can reduce physical endurance and mental acuity.^12,13,14^ Because functional limitations lead to lost independence, it is plausible that air pollution contributes to loss of independence; however, this has not been well-studied. Additionally, the loss of independence may better reflect the cumulative burden of air pollution in later life across multiple clinical and subclinical outcomes.

In this study, we leveraged a nationally representative cohort study to investigate associations of long-term concentrations of particulate matter air pollution (particulate matter less than 2.5 μm in diameter [PM_2.5]_ or ranging from 2.5 μm to 10 μm in diameter [PM_10-2.5_]), nitrogen dioxide (NO_2_), and ozone (O_3_) using estimates from a state-of-art spatiotemporal model with loss of independence.^15,16,17^ We focused on these pollutants because they are regulated by the Environmental Protection Agency (EPA). Given that different sources, such as agriculture, traffic, power generation, windblown dust, and wildfires, emit PM_2.5_ with distinct physical and chemical characteristics, we also evaluated PM_2.5_ levels from key emission sources.

## Methods

### Study Population

This cohort study used data from the Health and Retirement Study (HRS), a nationally representative sample of people older than 50 years in the US. HRS and our use of linkages of HRS residential addresses to environmental measures through the Environmental Predictors of Cognitive Health and Aging (EPOCH) study were approved by the University of Michigan institutional review board. Informed consent was obtained from all respondents, and we followed the Strengthening the Reporting of Observational Studies in Epidemiology (STROBE) reporting guideline.

HRS began recruiting in 1992 and added new birth cohorts every 6 years,^18^ with interviews biennially. For respondents who died or those unable or unwilling to be interviewed, the study surveyed a spouse, child, or another proxy.^19^

We studied all respondents with at least 2 interviews between 1998 and 2016, when our air pollution estimates were available. To assess the incidence, we included only respondents who had not previously reported receiving help with activities of daily living (ADL) or instrumental activities of daily living (IADL) or living in a nursing home at baseline. We also excluded respondents with missing data on pollution, independence, or confounders (eFigure 1 in Supplement 1).

### Identification of Incident Loss of Independence

We assessed new loss of independence at each interview for living respondents and in the last 3 months of life for respondents who had died. We classified loss of independence as newly receiving help in any ADL (ie, walking across the room, dressing, bathing or showering, eating, getting in or out of bed, and using the toilet) or IADL (ie, using the telephone, managing money, taking medications, grocery shopping, and preparing home meals) due to health or memory problems or moving to a nursing home.

In secondary analyses, we evaluated 2 mutually exclusive subcategories for lost independence: receiving assistance with at least 1 ADL item and receiving assistance solely with IADL or receiving no assistance but moving into a nursing home. We assumed that these 2 subtypes represented transitions from living independently to lost independence and receiving personal care.

### Air Pollution Assessment

We used spatiotemporal models to estimate PM_2.5_, PM_10-2.5_, NO_2_, and O_3_ concentrations at all respondent residential addresses from 1990 to 2016^20,21^ to assess mean exposures in the 10 years prior to each survey. These models incorporated regulatory monitor and research study network data, more than 300 geographic covariates, and spatial and temporal correlations to estimate mean concentrations at a 2-week resolution from 1999 to 2016 and an annual resolution from 1990 to 1999, when monitoring data for PM_2.5_ levels were more limited.^20,22^ With our interest in long-term exposures, we used year-round O_3_ level estimates instead of those for the warm season only. The cross-validation *R*^2^ for these models in most regions is greater than 0.80.^20,23^

We estimated source-specific concentrations of PM_2.5_ from agriculture, road traffic, nonroad traffic, energy from coal combustion, other energy, industry from coal combustion, other industry, wildfires, and windblown dust (eAppendix 1 in Supplement 1) by multiplying the total PM_2.5_ concentration at each residence by spatially resolved, fractional source-specific contributions of PM_2.5_.^24^ Briefly, these fractions were generated at a resolution of 0.5° × 0.625° from zero-out simulations in an atmospheric chemistry transport model (GEOS-Chem version 12.1.0).^24^ Although these models used 2017 emission data, we assumed that estimates reflected source contributions over the past decade given that 2017 emissions in the EPA National Emissions Inventory were strongly correlated with mean emissions from 2007 to 2017 for most sources (eFigure 2 in Supplement 1). We a priori focused on sources believed to have the most reliable emission information, as well as wildfires based on their increasing importance.

### Covariates

Detailed information about respondent demographic characteristics and health was collected during each follow-up interview. This included age, sex, self-reported race and ethnicity (Hispanic, non-Hispanic Black, non-Hispanic White, and non-Hispanic other [eg, American Indian or Alaska Native, Asian, and Pacific Islander]), marital status (married/partnered or unmarried/unpartnered), number of children (0, 1, 2, 3, or ≥4 children), educational attainment (<high school, General Educational Development test, high school, some college, ≥college), ownership of a primary residence, total household wealth, urbanicity (urban, suburban, or rural), and neighborhood social economic status (NSES) level. Race and ethnicity were assessed because there are known differences in the outcomes by race and ethnicity and systemic disinvestment in communities have resulted in differential pollution exposure by these same characteristics. We defined NSES using 11 US Census variables, including population income, housing, and occupation.^25^ Chronic diseases were self-reported at each interview, including diagnosis of CVDs (heart problems, stroke, or transient ischemic attack), chronic lung diseases except asthma, and diabetes. We also assessed the maximum normalized difference vegetation index near respondent homes (250 m and 1000 m buffers) for the year of the interview as a measure of green space. Annual mean temperatures were estimated for each respondent using state-level data from the Comparative Climactic Data center.

### Statistical Analysis

We used generalized estimating equation Poisson regression to examine the association of 10-year mean air pollution levels with loss of independence^26^ while accounting for person-level sampling weights, geographic strata, and sampling clusters. Our primary analysis estimated risk ratios (RRs) for loss of independence per 1-IQR increase in concentration of each pollutant from single-pollutant models. Then, in 2-pollutant models for PM_10-2.5_, NO_2_, and O_3_ levels, we additionally adjusted for total PM_2.5_ levels. Whereas 2-pollution models focused on source-specific PM_2.5_ levels, we included the sum of PM_2.5_ levels from all other sources. Lastly, we included all other pollutants (ie, PM_10-2.5_, NO_2_, and O_3_) in multipollutant models. We modeled source-specific PM_2.5_ levels as main effects instead of using the fractional source contributions as an effect modifier to allow for residential moves over time.

In all models, we adjusted for potential confounders, including baseline age, birth year, calendar date of interview, sex, race and ethnicity, marital status, number of children, educational attainment, ownership of primary residence at baseline, total household wealth at baseline fitted by natural splines with 5 degrees of freedom to account for potential nonlinearity, urbanicity, and NSES at each interview. We included multiple time-related variables because air pollution concentrations follow secular trends, age is a factor associated with high risk of loss of independence, and HRS recruited from a wide range of birth cohorts. Similarly, because SES is an important cofounder between air pollution and health outcomes, we adjusted for individual and neighborhood-level SES in our models. We also included an unpenalized thin-place regression spline with 10 degrees of freedom to flexibly adjust for residual spatial confounding.^27,28^

In secondary analyses, we estimated RRs per 1-μg/m^3^ increase in PM_2.5_ level. We also examined effect modification by baseline age category (≥75 or <75 years) and prevalent chronic disease (ie, CVD, chronic lung disease, or diabetes) on the multiplicative scale using interaction terms. To further explore mediation by chronic diseases, we adjusted for each disease in our models and observed changes in RRs. To learn more about potential mechanisms underlying these associations, we also explored associations with help required for ADL and IADL disability separately.

Using our observed RRs, we estimated the burden of incident loss of independence for the US population in 2015 attributable to any air pollutant robustly associated with a loss of independence following the Global Burden of Disease comparative risk assessment framework.^29^ We also evaluated the monetary cost associated with lost independence by estimating yearly costs per person for formal, informal, and nursing home care due to help for loss of independence, as described by Hurd et al^30^ and then multiplying our estimate of new individuals experiencing loss of independence from air pollution (eAppendix 2 in Supplement 1).

We performed several sensitivity analyses to evaluate the robustness of our findings. We tested for nonlinearity of our associations, used alternative mean exposure periods (1 years and 5 years), examined moving to nursing homes as an alternative outcome, and estimated hazard rate ratios using a Cox model. Because wildfires are temporally varying (eFigure 2 in Supplement 1), we also performed analyses using satellite-based estimates of wildfire-related PM_2.5_ levels from 2006 to 2017.^31,32^ Given that increased air temperatures promote the photochemical reaction of O_3_ and local vegetation is correlated with O_3_ levels,^33,34^ we adjusted our O_3_ models for green space and temperature. Finally, because individuals who need help may not receive it owing to social isolation, we restricted our analyses to participants who were married or partnered or had at least 1 child. We performed our data analyses from August 31 to October 15, 2023, in R statistical software version 4.2.1 (R Project for Statistical Computing) and SAS statistical software version 9.4 (SAS Institute). We determined statistical significance based on a *P* < .05 in a 2-sided test.

## Results

Our study included 25 314 participants (mean [SD] age, 61.1 [9.4] years at baseline; 11 208 male [44.3%]; 2741 Hispanic [10.8%], 4196 non-Hispanic Black [16.6%], and 17 675 non-Hispanic White [69.8%]). Nearly 40% of respondents (9985 individuals [39.4%]) experienced a new loss of independence during our mean (SD) 10.2 (5.5) years of follow-up (Table). Compared with participants who remained independent, respondents who lost their independence were more likely to be White and have lower levels of formal education, had a lower mean level of wealth, were more likely to have chronic diseases at baseline, and had been exposed to higher median levels of most pollutants.

**Table.** Study Population Characteristics^a^

Characteristic	Respondents, No. (%)			
	All (N = 25 314)	Remained independent (n = 15 329)	Lost independence (n = 9985)	
			Required ADL help (n = 4911)	Required IADL help only (n = 5074)
Follow-up, mean (SD), y	10.2 (5.5)	11.0 (5.7)	9.1 (4.7)	8.8 (4.7)
Age at baseline, mean (SD), y	61.1 (9.4)	57.5 (6.9)	66.9 (10.1)	66.5 (10.1)
Sex				
Male	11 208 (44.3)	6778 (44.2)	2180 (44.4)	2250 (44.3)
Female	14 106 (55.7)	8551 (55.8)	2731 (55.6)	2824 (55.7)
Race and ethnicity				
Hispanic	2741 (10.8)	1792 (11.7)	489 (10.0)	460 (9.1)
Non-Hispanic Black	4196 (16.6)	2636 (17.2)	741 (15.1)	819 (16.1)
Non-Hispanic White	17 675 (69.8)	10 401 (67.9)	3601 (73.3)	3673 (72.4)
Non-Hispanic Other^b^	702 (2.8)	500 (3.3)	80 (1.6)	122 (2.4)
Education				
<High school	5210 (20.6)	2209 (14.4)	1398 (28.5)	1603 (31.6)
GED	1189 (4.7)	702 (4.6)	217 (4.4)	270 (5.3)
High school	7558 (29.9)	4441 (29.0)	1569 (31.9)	1548 (30.5)
Some college	5938 (23.5)	3962 (25.8)	1001 (20.4)	975 (19.2)
≥College	5419 (21.4)	4015 (26.2)	726 (14.8)	678 (13.4)
Own primary residence	19 947 (78.8)	12 295 (80.2)	3810 (77.6)	3842 (75.7)
Baseline household wealth, mean (SD), $1000	249.8 (1042.9)	282.8 (1253.4)	206.3 (623.3)	192.3 (543.5)
NSES, mean (SD)^c^	0.23 (0.93)	0.18 (0.94)	0.29 (0.92)	0.31 (0.90)
Urbanicity				
Urban	13 166 (52.0)	8298 (54.1)	2441 (49.7)	2427 (47.8)
Suburban	5431 (21.5)	3192 (20.8)	1086 (22.1)	1153 (22.7)
Exurban	5652 (22.3)	3294 (21.5)	1133 (23.1)	1225 (24.1)
Chronic diseases at baseline				
CVD	4799 (19.0)	1979 (12.9)	1426 (29.0)	1394 (27.5)
Lung disease	1517 (6.0)	583 (3.8)	469 (9.5)	465 (9.2)
Diabetes	3633(14.4)	1776 (11.6)	999 (20.3)	858 (16.9)
10-y Mean air pollutants during follow-up, median (IQR)				
PM_2.5_, μg/m^3^				
Total	11.2 (9.5-13.3)	10.8 (9.4-13.1)	12.0 (10.4-14.2)	12.0 (10.4-14.3)
Agriculture	1.1 (0.6-1.7)	1.1 (0.6-1.6)	1.2 (0.7-1.8)	1.2 (0.7-1.8)
Road traffic	1.4 (1.1-1.7)	1.4 (1.1-1.7)	1.5 (1.2-1.8)	1.5 (1.2-1.8)
Nonroad traffic	0.4 (0.3-0.6)	0.4 (0.2-0.5)	0.4 (0.3-0.6)	0.4 (0.3-0.6)
Energy coal	0.8 (0.5-1.1)	0.8 (0.5-1.1)	0.8 (0.6-1.2)	0.8 (0.6-1.2)
Energy other	0.6 (0.4-0.7)	0.5 (0.4-0.7)	0.6 (0.5-0.8)	0.6 (0.5-0.8)
Industry coal	0.2 (0.2-0.3)	0.2 (0.2-0.3)	0.2 (0.2-0.3)	0.2 (0.2-0.3)
Industry other	0.9 (0.7-1.2)	0.9 (0.7-1.2)	1.0 (0.8-1.3)	1.0 (0.8-1.3)
Wildfires	1.0 (0.7-1.3)	1.0 (0.7-1.3)	1.1 (0.8-1.4)	1.1 (0.8-1.4)
Windblown dust	0.1 (0.0-0.1)	0.1 (0.0-0.1)	0.1(0.1-0.2)	0.1 (0.0-0.1)
Other pollutants				
PM_10-2.5_, μg/m^3^	8.9 (6.8-11.7)	8.8 (6.8-11.7)	9.1 (6.8-11.6)	9.0 (6.8-11.8)
NO_2_, ppb	9.0 (6.0-13.6)	8.5 (5.9-13.2)	9.8 (7.0-15.0)	9.9 (7.2-15.6)
O_3_, ppb	27.1 (24.8-28.6)	27.2 (25.0-28.6)	26.7 (24.2-28.1)	26.8 (24.3-28.3)

Abbreviations: ADL, activities of daily living; CVD, cardiovascular disease; GED, General Educational Development test; IADL, instrumental activities of daily living; NO_2_, nitrogen dioxide; NSES, neighborhood social economic status; O_3_, ozone; PM_2.5_, particulate matter less than 2.5 μm in diameter; PM_10-2.5_, particulate matter ranging from 2.5 μm to 10 μm in diameter; ppb, parts per billion.

^a^ Descriptive statistics are given for the study population from the Health and Retirement Study during follow-up from 1998 to 2016.

^b^ Other includes American Indian or Alaska Native, Asian, and Pacific Islander.

^c^ The 11 variables used to derive the NSES included income-related variables (ie, median household income and the percentage of households living under the poverty level and receiving public assistance, and the percentage of single-parent families), wealth-related variables (ie, the percentage of households that own their home; the percentage that receive interest, dividend, or rental income; and the median value of owner-occupied homes), education-related variables (ie, the percentage of persons with at least a high school degree and the percentage with at least a bachelor’s degree), and occupation-related variables (ie, the percentage unemployed and the percentage with a nonmanagerial occupation). NSES scores range from −4.34 to 2.68 in our study population, with higher scores indicating higher NSES.

**Figure 1.**
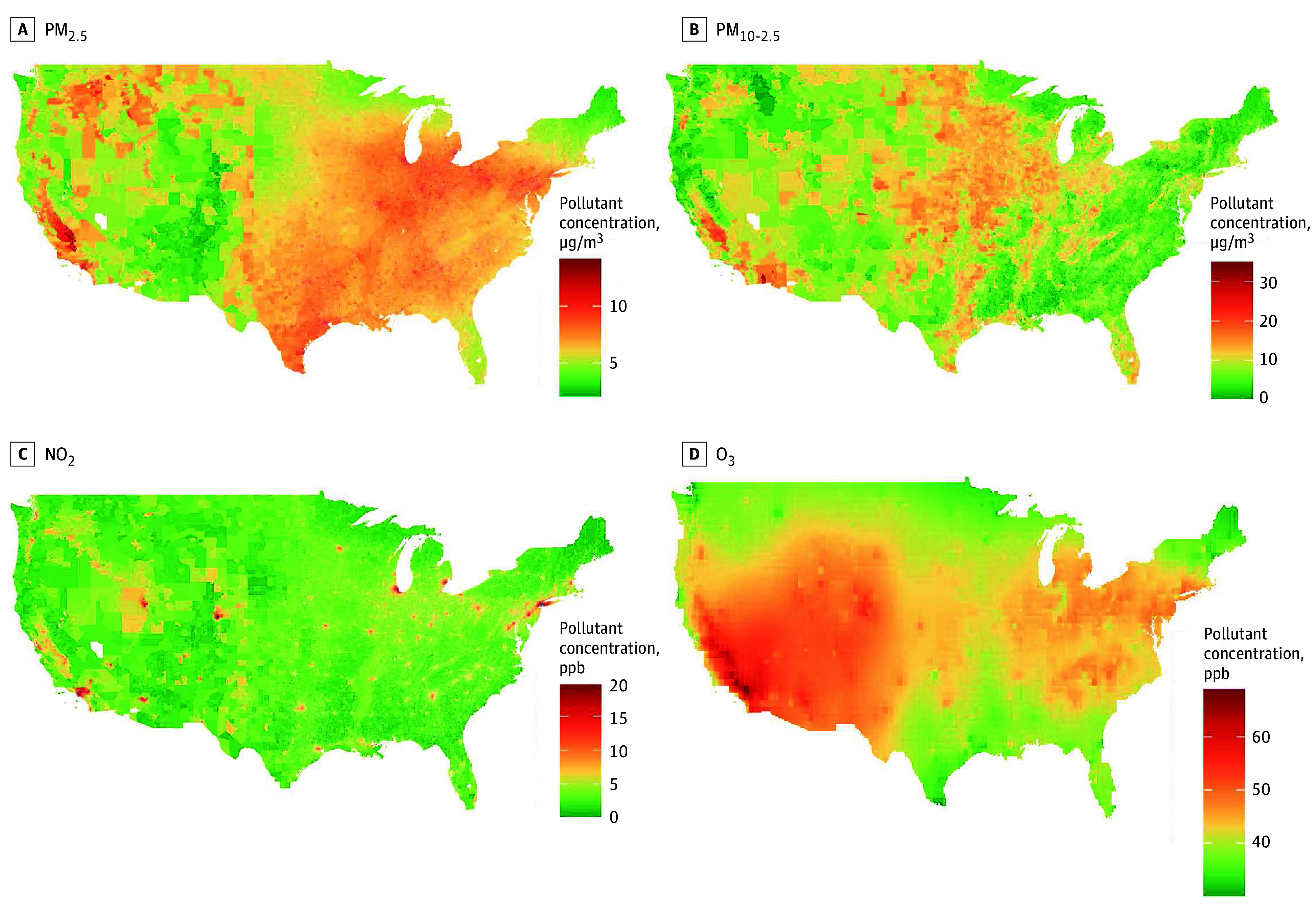
Spatial Distribution of Long-Term Concentrations of Air Pollutants in the US NO_2_ indicates nitrogen dioxide; O_3_, ozone; PM_2.5_, particulate matter less than 2.5 μm in diameter; PM_10-2.5_, particulate matter ranging from 2.5 μm to 10 μm in diameter; ppb, parts per billion.

The median (IQR) 10-year mean PM_2.5_ level was 11.2 (9.5-13.3) μg/m^3^ with the largest contributions from road traffic, agriculture, noncoal industry, and wildfires (Table). Total levels of PM_2.5_, PM_2.5_ from agriculture, traffic, and energy production all were higher in the Midwest and lower in the West (Figure 1; eFigure 3 in Supplement 1). In contrast, O_3_ and PM_2.5_ levels from industry, wildfires, and dust were higher in the West. There were shared spatial patterns, with relatively high correlations among PM_2.5_ levels from agriculture, traffic, and coal-related energy (eTable 1 in Supplement 1). For total PM_2.5_ levels, there was also a moderate positive correlation with NO_2_ and a negative correlation with O_3_ levels.

### Association of Air Pollution With Loss of Independence

**Figure 2.**
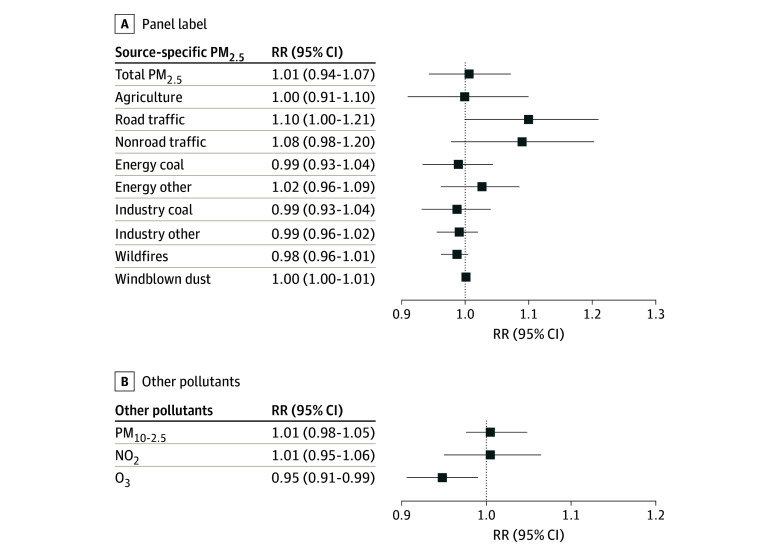
Association of Air Pollutants With Lost Independence Risk ratios are presented for the association of a 1-IQR increase in 10-year mean air pollutant levels with lost independence between 1998 and 2016 using multipollutant models in the Health and Retirement Study. All models were adjusted for the sum of particulate matter less than 2.5 μm in diameter (PM_2.5_) levels from all other sources/total PM_2.5_ levels, particulate ranging from 2.5 μm to 10 μm in diameter (PM_10-2.5_) levels, nitrogen dioxide (NO_2_) levels, ozone (O_3_) levels, baseline age, birth year, calendar date of interview, sex, race and ethnicity, marital status, number of children, educational attainment, ownership of the primary residence at baseline, total household wealth at baseline, time-varying urbanicity levels, time-varying neighborhood social economic status, and space with 10 degrees of freedom.

In single-pollutant models, we observed that a 1-IQR higher 10-year mean concentration of PM_2.5_ was associated with increased risk of loss of independence (RR, 1.05; 95% CI, 1.01-1.10) (eFigure 4 in Supplement 1). RRs per 1-IQR increase in 10-year mean concentrations in 9 source-specific PM_2.5_ ranged from 0.98 (95% CI, 0.96-1.01) for wildfires, which had no significant association, to the largest RRs, which were observed for road traffic–related (RR, 1.09; 95% CI, 1.03-1.16) and nonroad traffic–related (RR, 1.13; 95% CI, 1.03-1.24) PM_2.5_ levels. Notably, only road traffic–related PM_2.5_ levels remained robust to adjustment for PM_2.5_ from other sources in the 2-pollutant models and co-pollutants, with an RR of 1.10 (95% CI, 1.00-1.21) per 1-IQR increase in 10-year mean concentration in multipollutant models (Figure 2). The association with road traffic–related PM_2.5_ was similar to a difference in risk for a 2-year increase in age.

Among other pollutants (eFigure 4 in Supplement 1), NO_2_ levels were positively associated with an increased risk of loss of independence (RR per 1-IQR increase in 10-year mean concentration, 1.05; 95% CI, 1.01-1.08) in the single-pollutant model, which changed little with adjustment for total PM_2.5_ levels but became null in the multipollutant model (RR per 1-IQR increase in 10-year mean concentration, 1.01; 95% CI, 0.95-1.06) (Figure 2). In contrast, O_3_ levels per 1-IQR increase in 10-year mean concentration were observed to be robustly associated with lower risk of loss of independence (RR per 1-IQR increase in 10-year mean concentration, 0.94; 95% CI, 0.92-0.97) in the single-pollutant model. There were no associations for PM_10-2.5_ levels.

**Figure 3.**
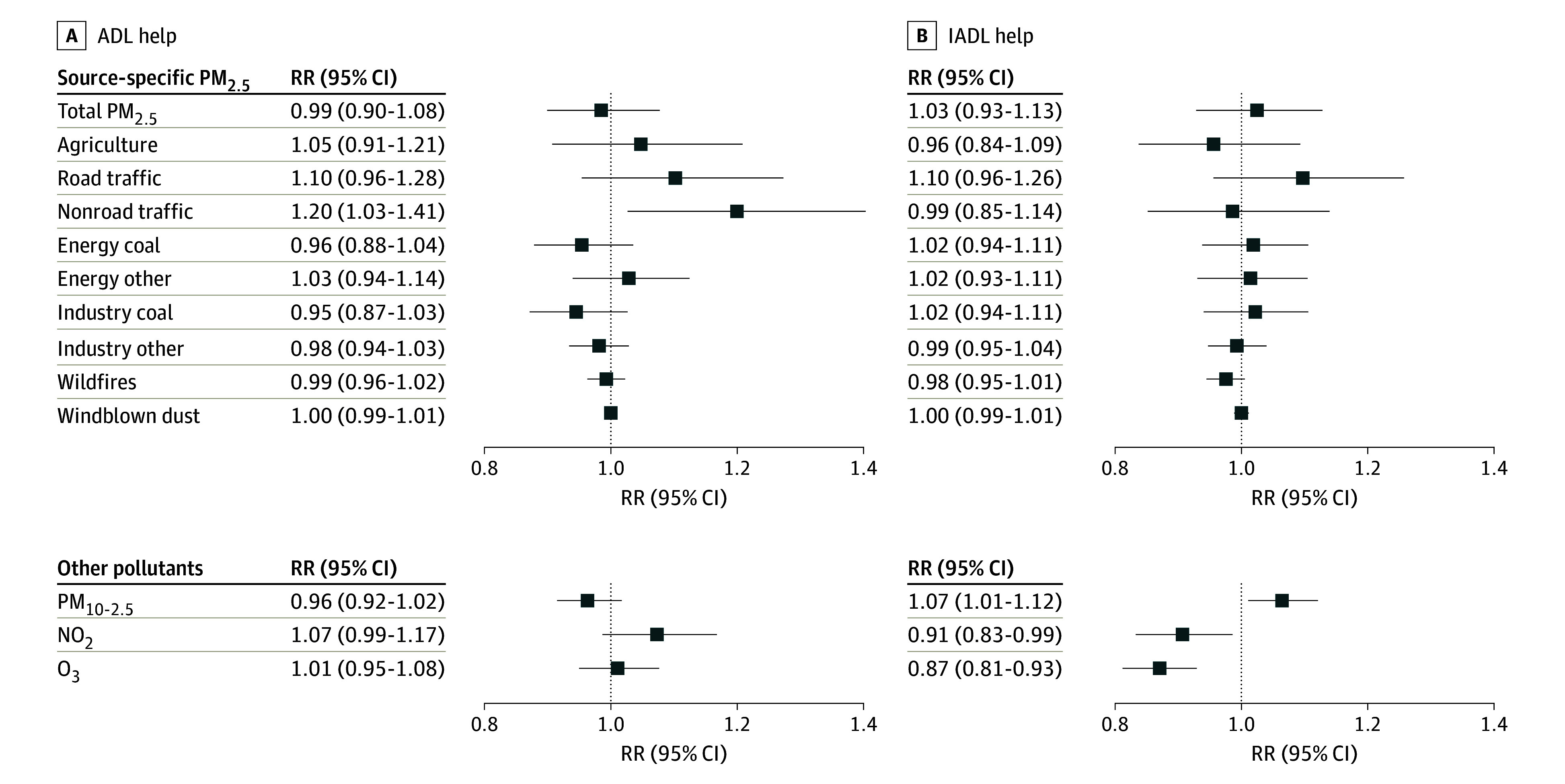
Association of Air Pollutants With Activities of Daily Life (ADL) Help Risk ratios are presented for the association of a 1-IQR increase in 10-year mean air pollutant levels with receiving ADL help and only receiving instrumental ADL (IADL) help between 1998 and 2016 using multipollutant models in the Health and Retirement Study. All models were adjusted for the sum of particulate matter less than 2.5 μm in diameter (PM_2.5_) levels from all other sources/total PM_2.5_ levels, particulate matter ranging from 2.5 μm to 10 μm in diameter (PM_10-2.5_) levels, nitrogen dioxide (NO_2_) levels, ozone (O_3_) levels, baseline age, birth year, calendar date of interview, sex, race and ethnicity, marital status, number of children, educational attainment, ownership of the primary residence at baseline, total household wealth at baseline, time-varying urbanicity levels, time-varying neighborhood social economic status, and space with 10 degrees of freedom.

In secondary analyses, we found that the main contribution for all observed associations was the need for ADL help (eFigure 5 in Supplement 1; Figure 3). Higher levels of PM_2.5_ from road traffic, nonroad traffic, and NO_2_ were robustly associated with higher risks of ADL help, and higher levels of O_3_ were associated with lower risks of ADL help. No associations were found with help involving only IADL. Most RRs were generally higher among participants younger than 75 years at baseline (eTable 2 in Supplement 1) and participants with prevalent CVD or diabetes but not chronic lung disease (eTable 3 in Supplement 1). RRs were not reduced after adjustment for CVD, chronic lung diseases, or diabetes (eTable 4 in Supplement 1). Given that robust associations with loss of independence were found only for PM_2.5_ levels from road and nonroad traffic, we focused our burden calculations on these sources, estimating that nearly 729 727 new cases per year of loss of independence may have been attributable to traffic-related PM_2.5_ levels in the US, assuming our associations are unbiased. Given that the yearly cost to receive help for lost independence was $16 028 per person, we estimated that the total cost of individuals newly losing independence due to exposure to traffic-related air pollution was $11.7 billion per year.

Our primary findings remained relatively robust in sensitivity analyses. For example, associations for most air pollutants were approximately linear, with only the suggestion of lower RRs at higher concentrations for total PM_2.5_, PM_2.5_ from industry coal, and NO_2_ (eFigure 6 in Supplement 1). Our primary findings were robust to using different exposure windows (eFigure 7 in Supplement 1), fitting with a Cox model (eTable 5 in Supplement 1), restriction to individuals not socially isolated (eTable 6 in Supplement 1), additional adjustment for green space or temperature (eTable 7 in Supplement 1), the use of time-varying wildfire-related PM_2.5_ levels (eTable 8 in Supplement 1), and investigation of moving to nursing homes (eTable 9 in Supplement 1).

## Discussion

In this national cohort study, we found higher risks of loss of independence due to health or memory problems among older adults who experienced higher long-term concentrations of air pollutants. Notably, there were differences across air pollutant types and emission sources, with the highest RRs and most robust associations after adjustment for PM_2.5_ levels from road and nonroad traffic. In contrast, lower O_3_ concentrations were associated with higher risks of loss of independence. Receiving help for ADL was the main contributor to all associations, reflecting more severe declines in physical and cognitive function. We found no evidence of mediation, but RRs in the association of air pollution with loss of independence were higher in individuals with chronic diseases, including CVD and diabetes.

This study contributes to the literature by newly evaluating associations between long-term exposure to air pollution and help for health or memory problems at older ages, which may be associated with physical or cognitive functional losses due to air pollution’s impacts on chronic disease development. Although related work has investigated associations with self-reported difficulties in ADL^12,35,36^ and performance-based tests^14,37,38,39^ alone, to our knowledge, our outcome is novel in its capture of more severe dysfunction that requires personal care for ADL and IADL. This may make this research more directly informative of the economic burdens associated with air pollution in older adults. If the observed associations were estimated without bias, nearly 729 727 new cases of loss of independence per year were attributable to traffic-related air pollution in the US, with a cost of $11.7 billion per year.

Although no other studies to our knowledge have assessed long-term exposure to pollution and lost independence, our findings are consistent with other research on physical and cognitive functional declines. For example, the Chinese Longitudinal Healthy Longevity Study^40^ documented a cross-sectional association between an air pollution index and higher risks of physical functional declines measured by difficulties in performing ADL and longitudinal associations between increased PM_2.5_ levels and a reduction in hand grip strength and balance ability.^37^ Also in China, there has been evidence that pollution from household heating is a risk factor for functional declines.^36,38^ For example, in the China Health and Retirement Longitudinal Study,^36^ a sister study of HRS, burning of coal, wood, or crop residue for heating was associated with higher risks of ADL and IADL limitations compared with clean heating energy. A 2021 systematic review^41^ also found associations between PM_2.5_ levels and cognitive declines. In 2021, a systematic review and meta-analysis^42^ also suggested that PM_2.5_ and NO_2_ levels were risk factors associated with dementia.

Notably, we found evidence that long-term exposures to pollutants from traffic (ie, PM_2.5_ from road and nonroad traffic and NO_2_ levels) were more consistently associated with a need for help due to health or memory problems. This is plausible given that PM_2.5_ from traffic contains harmful chemicals, including heavy metals, black carbon, and polycyclic aromatic hydrocarbons,^43^ that may translocate throughout the body in the bloodstream due to the particles’ small sizes (<0.1 μm).^44^ Our finding of increased toxic effects of traffic-related pollution is also consistent with findings from the Chicago Health and Aging Project^14^ that NO_2_ concentrations were associated with faster declines in basic physical functions, a common precursor of loss of independence. The finding is also consistent with a broader literature that suggests that compared with other sources, traffic-related air pollution may be associated with higher risks of chronic conditions, including CVD,^8,45,46,47^ respiratory disease,^48^ diabetes,^49^ and dementia,^50^ although we did not find a robust association of traffic-related PM_2.5_ levels with dementia in the HRS.^51^

Counter to our hypothesis, we found that lower O_3_ concentrations were associated with higher risks of loss of independence. Sensitivity analyses showed that these associations were robust to adjustment for temperature and green space. Similarly, these findings could not be explained by negative correlations with other measured pollutants. However, it is possible that these unexpected findings reflect the conversion of O_3_ into other highly oxidized pollutants in the atmosphere that may be important for health but are unmeasured. Notably, the very small literature on O_3_ levels and functional declines is inconsistent.^41,52^ While a Korean cohort study^53^ reported that higher O_3_ levels were associated with greater odds of frailty, higher O_3_ concentrations were associated with fewer disability days in a Toronto cohort.^52^ In another cross-sectional study of O_3_ levels and cognitive function,^54^ associations with executive function were observed only when levels were greater than 49 ppb, suggesting a complicated and nonlinear association of O_3_ with cognition. Ultimately, however, more evidence is needed to understand the association of O_3_ levels with loss of independence.

To our knowledge, this is the first study to directly evaluate associations between long-term exposure to air pollution and loss of independence as assessed by the use of informal or formal care for health or memory problems in later life. Relatedly, this research was conducted as a nationally representative study in the US, in which multiple air pollutants were estimated at respondent residential addresses and were at lower levels than in previous, related studies.^12,35,37,38^ Compared with using specific components as tracers for sources, our estimates more directly reflect sources. They also include primary emissions and secondary pollution generated from chemical reactions that would not exist if that source were removed.

### Limitations

Our study has limitations that merit attention. First, our emphasis was on the loss of independence as measured by the use of care rather than the need for care. This raises concerns that our outcome may be associated with factors, like SES, that estimate the use of care and lead to outcome misclassification. However, we incorporated formal and informal care and included detailed adjustments for social supports and SES estimated at individual and neighborhood levels. Additionally, our findings were robust to excluding respondents who were socially isolated. We cannot completely rule out the possibility that our observed associations are attributable to unmeasured confounding by other factors, like access to nutritious food, but adjustment for SES and urbanicity and a flexible adjustment for place should help mitigate this risk. Moreover, although we assumed an irreversible pathway from living independently to loss of independence in our analysis, the truth may be more complex, with the possibility of recovering independence over time.

Regarding our exposure estimation, it is notable that our estimates of source-specific PM_2.5_ levels have limited temporal resolution. However, there is high temporal stability of emissions over the past decade for most sources, and our findings were robust to more extensive time-varying estimates of wildfire-related PM_2.5_ levels.^31^ Additionally, there is a risk of healthy survivor bias in a prospective study of older adults.^55,56^ If participants who remained in the study were less susceptible to pollution, then our associations would be biased toward the null.^57^ Our use of proxy respondents, information on respondents who died, and time-varying survey weights should mitigate selection bias, although higher RRs for younger respondents may still indicate healthy selection bias.

## Conclusions

This cohort study found that higher long-term air pollution concentrations were associated with increased risks of lost independence due to health or memory problems among older adults, with especially high RRs and associations robust to adjustment found for pollution from traffic. These findings suggest that reducing exposures may be associated with diversion or delay of the need for care at older ages and enhanced ability to live independently.

## Notes

Supplement 1. **eAppendix 1.** Description of 9 emission sources **eAppendix 2.** Estimation of the burden of incident loss of independence attributable to air pollution for the US population in 2015 **eFigure 1.** Schematic flowchart of the study population selection **eFigure 2.** Correlations between primary source-specific PM2.5 emissions from 2017 and 10-year mean primary source-specific PM2.5 emissions from 2007-2017 for each state in the US **eFigure 3.** Spatial distribution of long-term concentrations of source-specific PM2.5 across the US **eFigure 4.** Risk ratios for lost independence between 1998 and 2016 corresponding to an IQR increase in 10-year mean of air pollutants in Health and Retirement Study **eFigure 5.** Risk ratios for receiving ADL help and only receiving IADL help between 1998 and 2016 associated with an IQR increase in 10-year mean of air pollutants in the Health and Retirement Study **eFigure 6.** Smoothing curves of the associations between air pollutants and adjusted RR (95% CI) of loss of independence using GEE Poisson regression with natural splines **eFigure 7.** Risk ratios (95% CIs) for lost independence between 1998 and 2016 corresponding to an IQR increase in air pollutants concentrations in single-pollutant models with different exposure mean periods **eTable 1.** Correlations among 10-year mean air pollutants for respondents of the Health and Retirement Study (1998-2016) **eTable 2.** Risk ratios for lost independence between 1998 and 2016 associated with an IQR increase in 10-year mean of air pollutants in the Health and Retirement Study modified by baseline age **eTable 3.** Risk ratios for lost independence between 1998 and 2016 associated with an IQR increase in 10-year mean of air pollutants in the Health and Retirement Study modified by common chronic diseases **eTable 4.** Risk ratios for lost independence between 1998 and 2016 associated with an IQR increase in 10-year mean of air pollutants in the Health and Retirement Study further adjusting for common chronic diseases **eTable 5.** Hazard ratios for lost independence between 1998 and 2016 associated with an IQR increase in 10-year mean of air pollutants in the Health and Retirement Study **eTable 6.** Risk ratios for lost independence between 1998 and 2016 associated with an IQR increase in 10-year mean of air pollutants in the Health and Retirement Study modified when restricting to those not socially isolated **eTable 7.** The risk of loss of independence per quartile increases in 10-year mean of O3 in the Health and Retirement Study **eTable 8.** Risk ratios for lost independence in the Health and Retirement Study associated with an IQR increase in mean concentrations of PM2.5 from wildfires estimated with time-fixed emission data (primary) and wildfires estimated with time-varying emission data (2006 to 2016) with different mean periods and baseline **eTable 9.** Risk ratios for moving to nursing homes between 1998 and 2016 associated with an IQR increase in 10-year mean of air pollutants in the Health and Retirement Study

Supplement 2.
Data Sharing Statement
