# Supplementary material for: Source-Specific Air Pollution and Loss of Independence in Older Adults Across the US
Source: JAMA Netw Open. 2024 Jun 28;7(6):e2418460. doi: 10.1001/jamanetworkopen.2024.18460 (PMC11214115; doi:10.1001/jamanetworkopen.2024.18460)
Supplement: Supplement 1. — eAppendix 1. Description of 9 emission sources eAppendix 2. Estimation of the burden of incident loss of independence attributable to air pollution for the US population in 2015 eFigure 1. Schematic flowchart of the study population selection eFigure 2. Correlations between primary source-specific PM2.5 emissions from 2017 and 10-year mean primary source-specific PM2.5 emissions from 2007-2017 for each state in the US eFigure 3. Spatial distribution of long-term concentrations of source-specific PM2.5 across the US eFigure 4. Risk ratios for lost independence between 1998 and 2016 corresponding to an IQR increase in 10-year mean of air pollutants in Health and Retirement Study eFigure 5. Risk ratios for receiving ADL help and only receiving IADL help between 1998 and 2016 associated with an IQR increase in 10-year mean of air pollutants in the Health and Retirement Study eFigure 6. Smoothing curves of the associations between air pollutants and adjusted RR (95% CI) of loss of independence using GEE Poisson regression with natural splines eFigure 7. Risk ratios (95% CIs) for lost independence between 1998 and 2016 corresponding to an IQR increase in air pollutants concentrations in single-pollutant models with different exposure mean periods eTable 1. Correlations among 10-year mean air pollutants for respondents of the Health and Retirement Study (1998-2016) eTable 2. Risk ratios for lost independence between 1998 and 2016 associated with an IQR increase in 10-year mean of air pollutants in the Health and Retirement Study modified by baseline age eTable 3. Risk ratios for lost independence between 1998 and 2016 associated with an IQR increase in 10-year mean of air pollutants in the Health and Retirement Study modified by common chronic diseases eTable 4. Risk ratios for lost independence between 1998 and 2016 associated with an IQR increase in 10-year mean of air pollutants in the Health and Retirement Study further adjusting for common chronic diseases eTable 5. H [file jamanetwopen-e2418460-s001.pdf]

Supplemental Online Content

Zhang B, Mendes de Leon CF, Langa KM, et al. Source-specific air pollution and loss of independence in older adults across the US. *JAMA Netw Open*. 2024;7(6):e2418460. doi:10.1001/jamanetworkopen.2024.18460

eAppendix 1. Description of 9 emission sources

eAppendix 2. Estimation of the burden of incident loss of independence attributable to air pollution for the US population in 2015

eFigure 1. Schematic flowchart of the study population selection

eFigure 2. Correlations between primary source-specific PM2.5 emissions from 2017 and 10-year mean primary source-specific PM2.5 emissions from 2007-2017 for each state in the US

eFigure 3. Spatial distribution of long-term concentrations of source-specific PM2.5 across the US

eFigure 4. Risk ratios for lost independence between 1998 and 2016 corresponding to an IQR increase in 10-year mean of air pollutants in Health and Retirement Study

eFigure 5. Risk ratios for receiving ADL help and only receiving IADL help between 1998 and 2016 associated with an IQR increase in 10-year mean of air pollutants in the Health and Retirement Study

eFigure 6. Smoothing curves of the associations between air pollutants and adjusted RR (95% CI) of loss of independence using GEE Poisson regression with natural splines

eFigure 7. Risk ratios (95% CIs) for lost independence between 1998 and 2016 corresponding to an IQR increase in air pollutants concentrations in single-pollutant models with different exposure mean periods

eTable 1. Correlations among 10-year mean air pollutants for respondents of the Health and Retirement Study (1998-2016)

eTable 2. Risk ratios for lost independence between 1998 and 2016 associated with an IQR increase in 10-year mean of air pollutants in the Health and Retirement Study modified by baseline age

eTable 3. Risk ratios for lost independence between 1998 and 2016 associated with an IQR increase in 10-year mean of air pollutants in the Health and Retirement Study modified by common chronic diseases

eTable 4. Risk ratios for lost independence between 1998 and 2016 associated with an IQR increase in 10-year mean of air pollutants in the Health and Retirement Study further adjusting for common chronic diseases

eTable 5. Hazard ratios for lost independence between 1998 and 2016 associated with an IQR increase in 10-year mean of air pollutants in the Health and Retirement Study

© 2024 Zhang B et al. *JAMA Network Open.*

eTable 6. Risk ratios for lost independence between 1998 and 2016 associated with an IQR increase in 10-year mean of air pollutants in the Health and Retirement Study modified when restricting to those not socially isolated

eTable 7. The risk of loss of independence per quartile increases in 10-year mean of O3 in the Health and Retirement Study

eTable 8. Risk ratios for lost independence in the Health and Retirement Study associated with an IQR increase in mean concentrations of PM2.5 from wildfires estimated with time-fixed emission data (primary) and wildfires estimated with time-varying emission data (2006 to 2016) with different mean periods and baseline

eTable 9. Risk ratios for moving to nursing homes between 1998 and 2016 associated with an IQR increase in 10-year mean of air pollutants in the Health and Retirement Study

This supplemental material has been provided by the authors to give readers additional information about their work.

© 2024 Zhang B et al. *JAMA Network Open.*

eAppendix 1. Description of Nine Emission Sources[1]

Agriculture - Includes manure management, soil fertilizer emissions, rice cultivation, enteric fermentation, and other agriculture

Road Traffic - Includes cars, motorcycles, heavy and light duty trucks and buses

Non-road Traffic - Includes rail, domestic navigation, other transportation

Coal Combustion for Energy Production - Includes electricity and heat production, fuel production and transformation, oil and gas fugitive/flaring, and fossil fuel fires

Other Energy Production - Includes electricity and heat production, fuel production and transformation, oil and gas fugitive/flaring, and fossil fuel fires

Coal Combustion for Industry - Includes industrial combustion (iron and steel, non-ferrous metals, chemicals, pulp and paper, food and tobacco, non-metallic minerals, construction, transportation equipment, machinery, mining and quarrying, wood products, textile and leather, and other industry combustion) and non-combustion industrial processes and product use (cement production, lime production, other minerals, chemical industry, metal production, food, beverage, wood, pulp, and paper, and other non-combustion industrial emissions)

Other Industry - Includes industrial combustion (iron and steel, non-ferrous metals, chemicals, pulp and paper, food and tobacco, non-metallic minerals, construction, transportation equipment, machinery, mining and quarrying, wood products, textile and leather, and other industry combustion) and non-combustion industrial processes and product use (cement production, lime production, other minerals, chemical industry, metal production, food, beverage, wood, pulp, and paper, and other non-combustion industrial emissions)

Wildfires - Includes deforestation, boreal forest, peat, savannah, and temperate forest fires (from the GFED fires inventory)

Windblown Dust - (from the DEAD dust model)

© 2024 Zhang B et al. *JAMA Network Open.*

eAppendix 2. Estimation of the burden of incident loss of independence attributable to air pollution for the US population in 2015

Step 1.

We used the average of the minimum and fifth percentiles of the respective distribution of each air pollutant found to be robustly associated with loss of independence in Model 3 as the theoretical minimum risk exposure level (TMREL). Concentrations at or below this level are considered to reflect background concentrations and the baseline risks of loss of independence without any added anthropogenic pollution (i.e., RR of 1).

Step 3.

We used the 10-year average tract-level concentrations of air pollutants over 10 years before 2015 to estimate the RRs of loss of independence attributable to the air pollution in each census tract.

Step 4.

Then, we estimated the tract-level attributable fraction (AF) of incident dementia due to air pollution exposure.

$$AF_{pollutant,tract} = 1 - 1/RR_{pollutant,tract}$$

Step 5.

We obtained the total number of loss of independence cases that could be attributed to each air pollutant by summing the product of all tract-level AF, risks of loss of independence and population in 2015 among those older than 50 years old over the continental US. Given expected differences in risks of loss of independence in different subpopulations, we estimated the risks separately for each region (Northeast, Midwest, South, and West), race/ethnicity, and age group (50-64, 65-74, 75-84, >85) separately using HRS data.

Step 6.

Finally, we estimated the yearly costs per person associated with help for people losing independence on the basis of self-reported formal, informal, and nursing home care among HRS respondents interviewed during the 2016 wave. We followed the method described by Hurd's study to calculate the cost due to the need for help [2]. With these individual costs and the number of cases attributable to traffic-related $PM_{2.5}$ we obtained in Step 5, we calculated the total cost of loss of independence in 2015.

© 2024 Zhang B et al. *JAMA Network Open.*

Figures

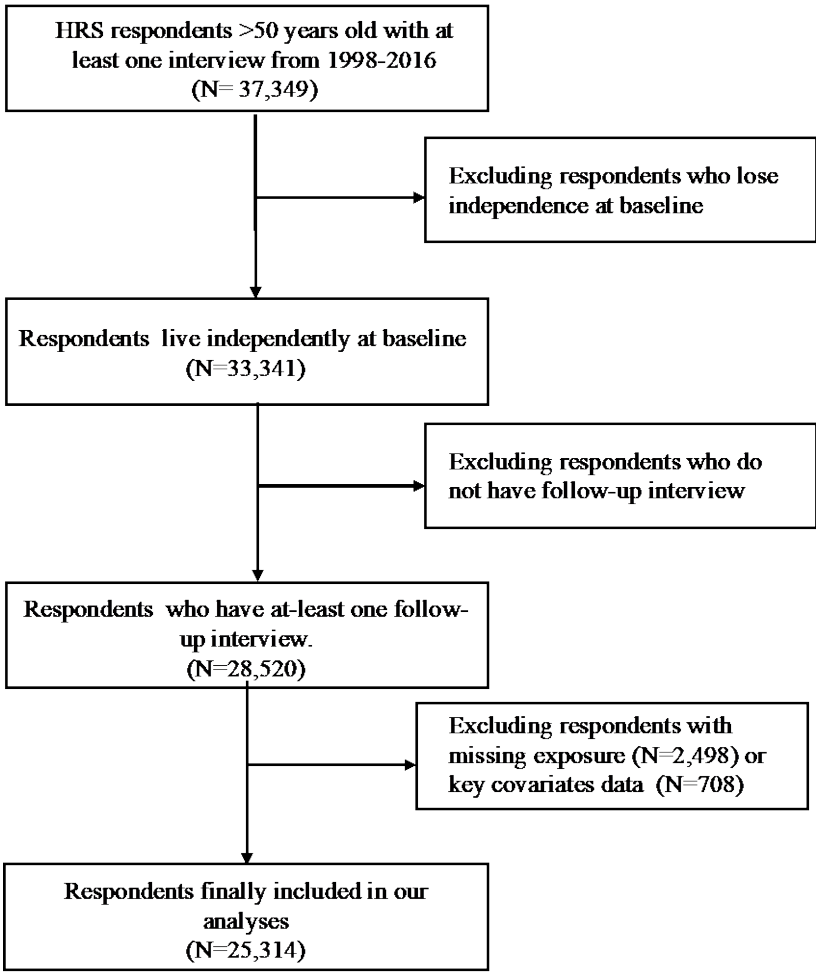

eFigure 1. Schematic flowchart of the study population selection.

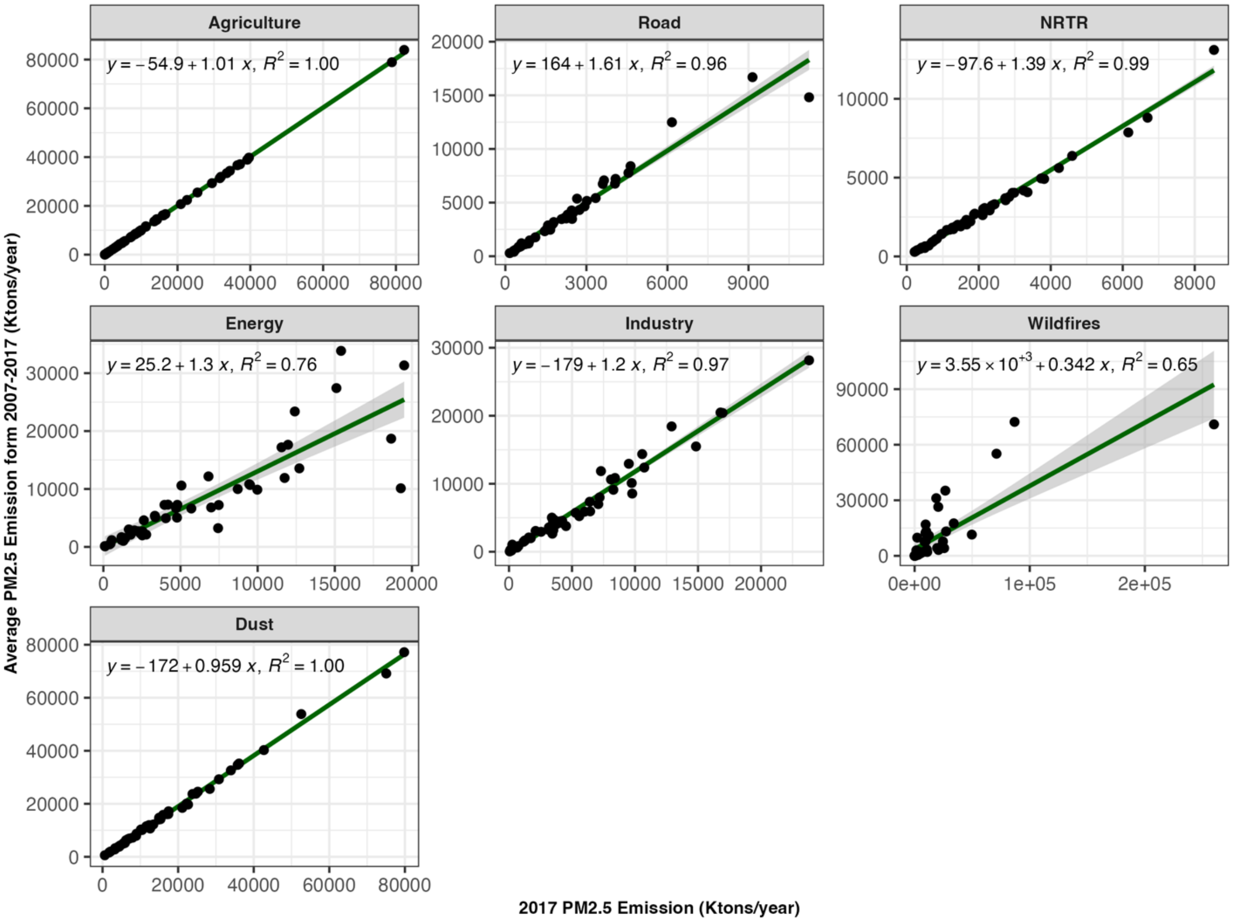

eFigure 2. Correlations between primary source-specific $PM_{2.5}$ emissions from 2017 and 10-year average primary source-specific $PM_{2.5}$ emissions from 2007-2017 for each state in the United States.

© 2024 Zhang B et al. *JAMA Network Open.*

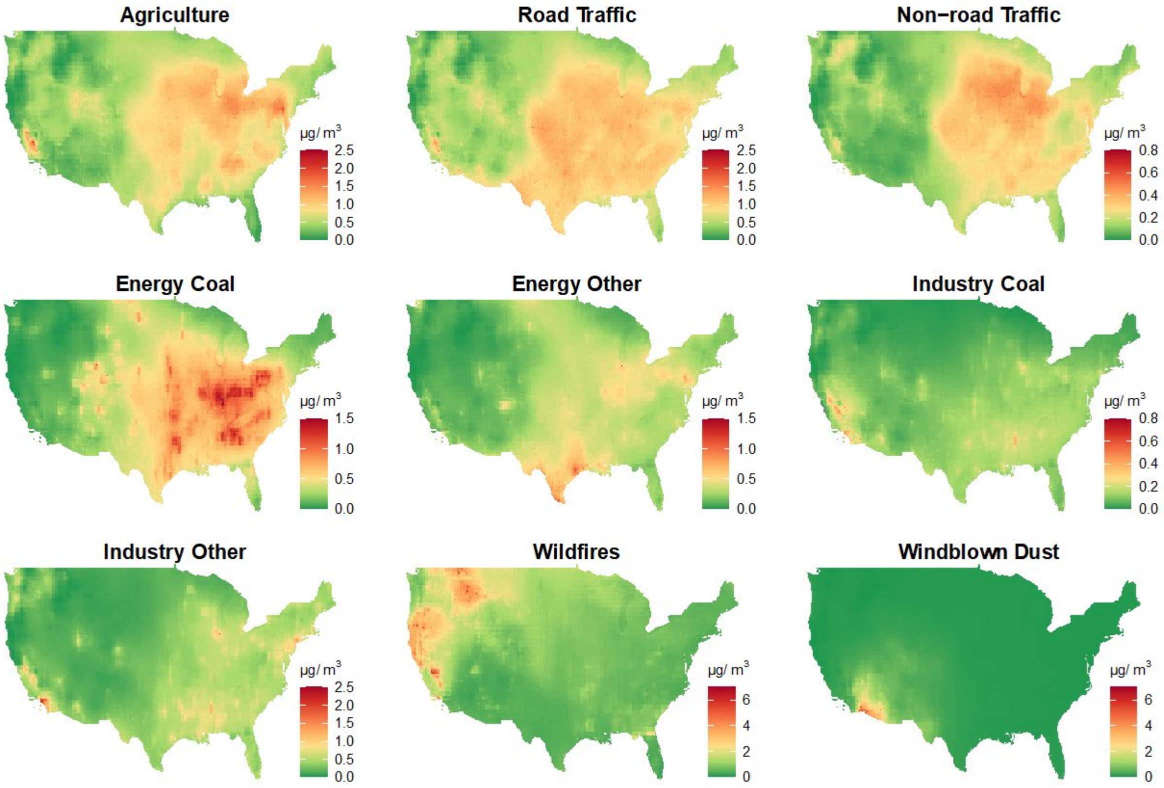

eFigure 3. Spatial distribution of long-term concentrations of source-specific $PM_{2.5}$ across the United States.

© 2024 Zhang B et al. *JAMA Network Open.*

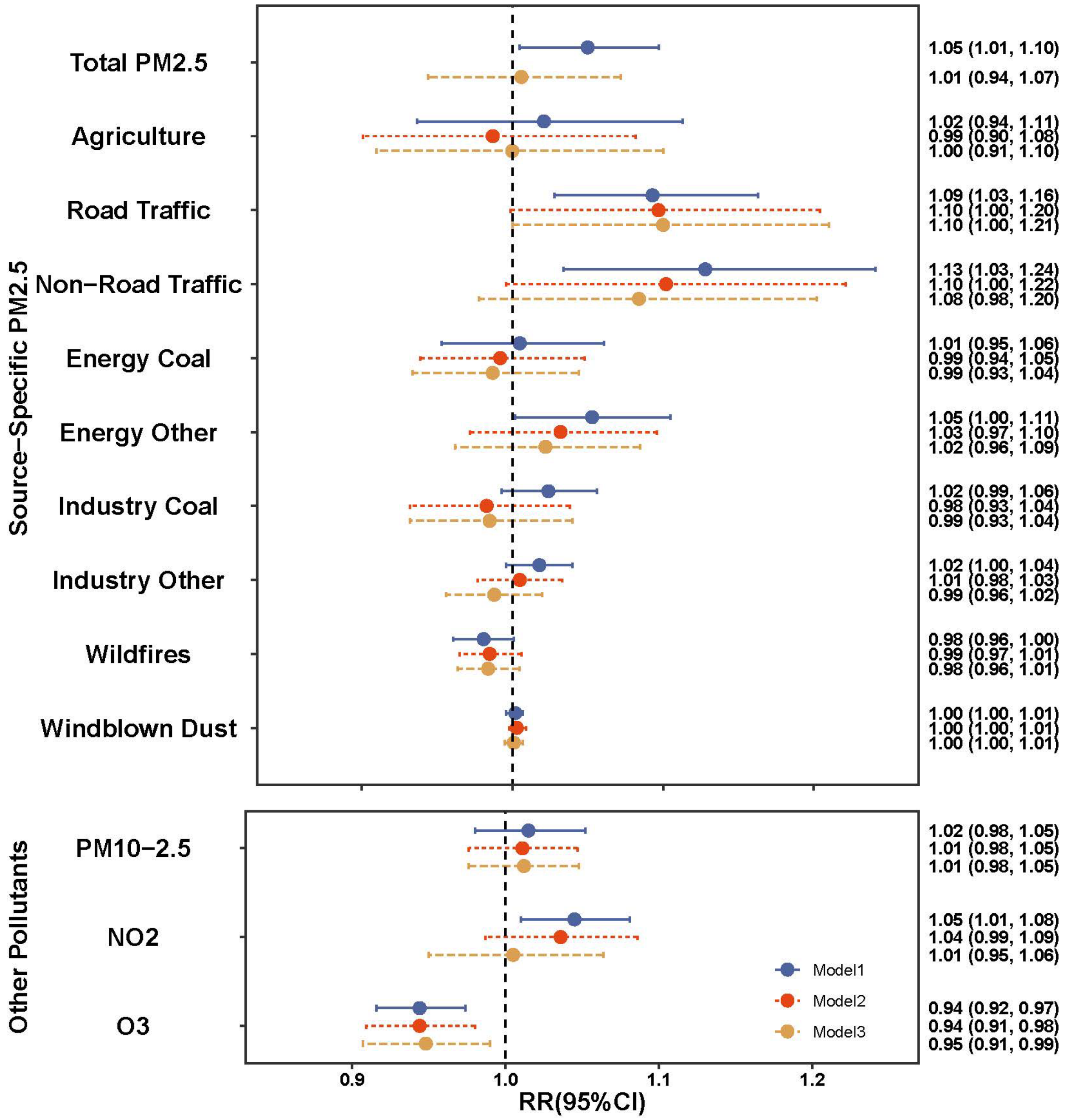

eFigure 4. Risk ratios for lost independence between 1998 and 2016 corresponding to an IQR increase in 10-year average of air pollutants, in Health and Retirement Study.

a. Model 1: Single-pollutant model; Model 2 (Two-pollutant model): Model 1 for source-specific $PM_{2.5}$ + the sum of $PM_{2.5}$ from all other source/ Model 1 for other pollutants + total $PM_{2.5}$; Model 3 (Multi-pollutant model): Model 2+ $PM_{10\text{-}2.5}$, NO_2, O_3.
b. All models were adjusted for baseline age, birth year, calendar date of interview, sex, race/ethnicity, marital status, number of children, educational attainment, ownership of the primary residence at baseline, total household wealth at baseline, time-varying urbanicity levels, time-varying NSES, and space with 10 df.

© 2024 Zhang B et al. *JAMA Network Open.*

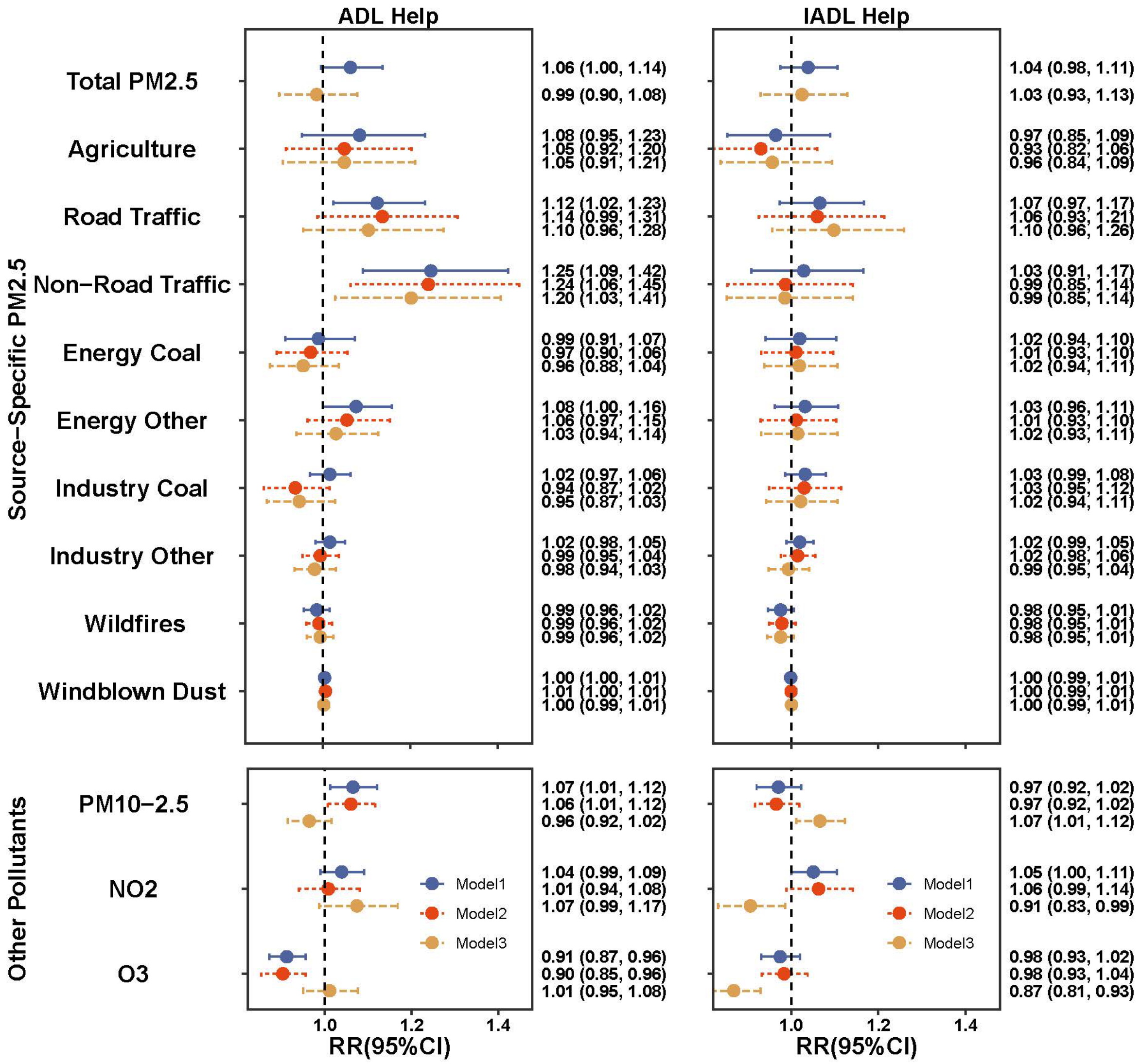

eFigure 5. Risk ratios for receiving ADL help and only receiving IADL help between 1998 and 2016 associated with an IQR increase in 10-year average of air pollutants in the Health and Retirement Study.

a. Model 1: Single-pollutant model; Model 2 (Two-pollutant model): Model 1 for source-specific $PM_{2.5}$ + the sum of $PM_{2.5}$ from all other source/Model 1 for other pollutants + total $PM_{2.5}$; Model 3 (Multi-pollutant model): Model 2+ $PM_{10-2.5}$, NO_2, O_3.
b. All models were adjusted for baseline age, birth year, calendar date of interview, sex, race/ethnicity, marital status, number of children, educational attainment, ownership of the primary residence at baseline, total household wealth at baseline, time-varying urbanicity levels, time-varying NSES, and space with 10 df.

© 2024 Zhang B et al. *JAMA Network Open.*

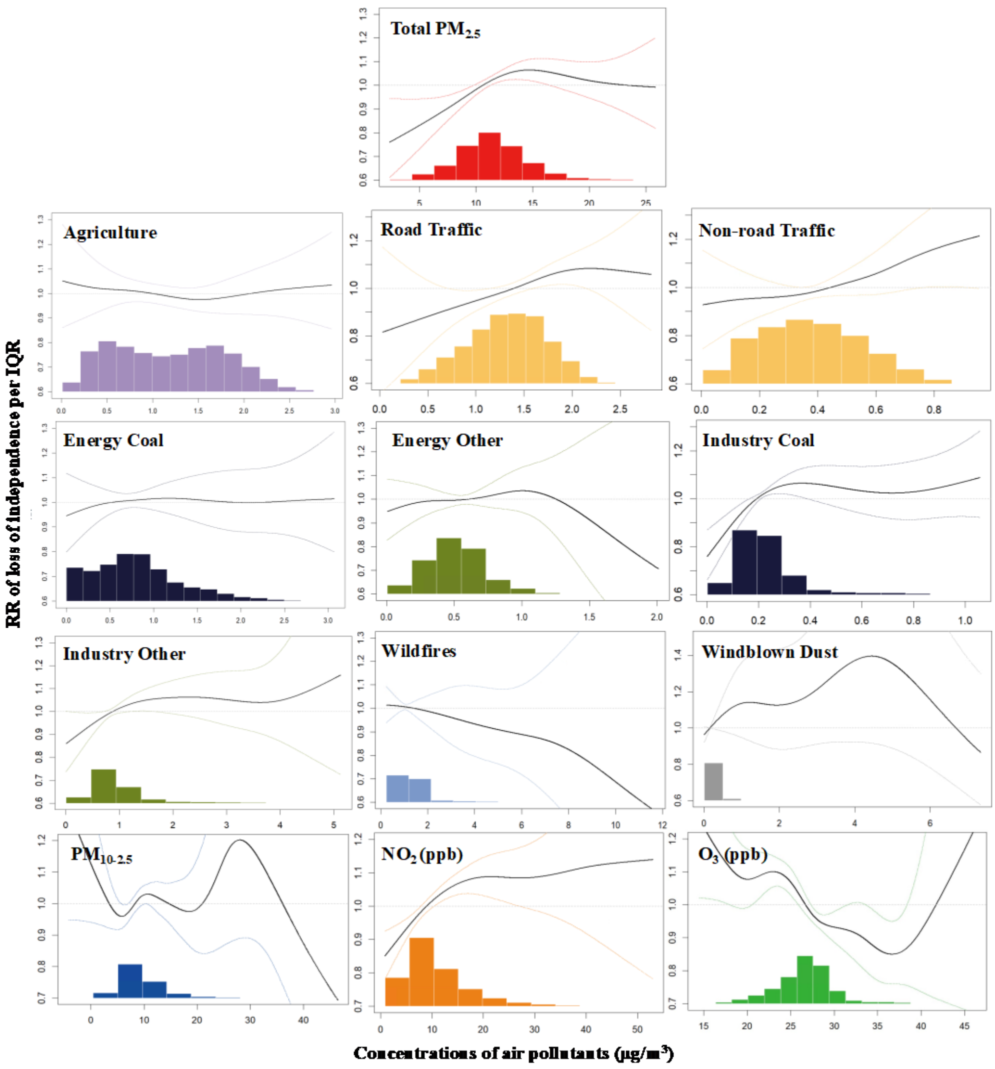

eFigure 6. Smoothing curves of the relationships between air pollutants and adjusted RR (95% CI) of loss of independence using GEE Poisson regression with natural splines. The models were adjusted all the covariates included in our main models.

© 2024 Zhang B et al. *JAMA Network Open.*

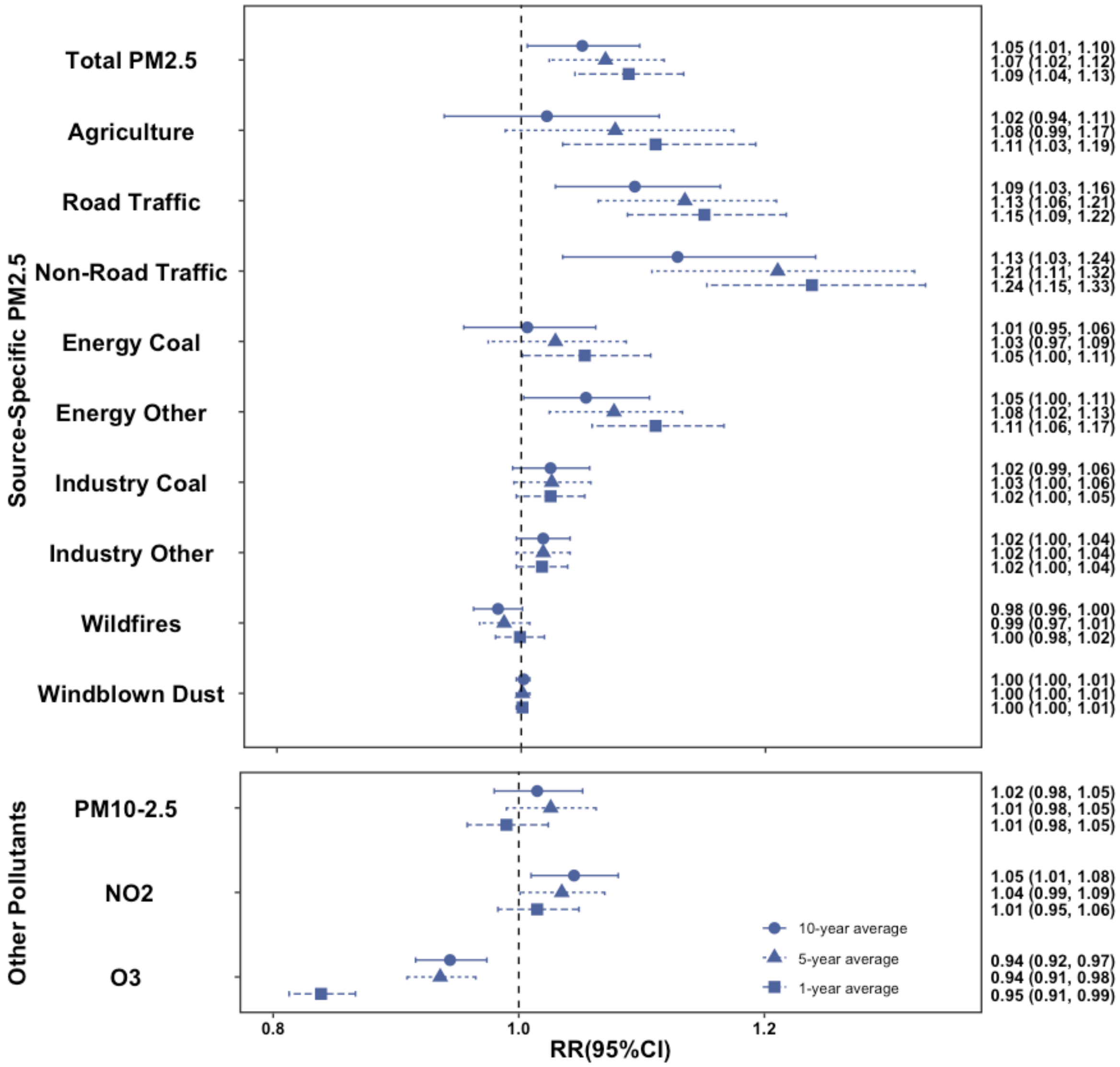

eFigure 7. Risk ratios (95% confidence intervals) for lost independence between 1998 and 2016 corresponding to an IQR increase in air pollutants concentrations in single pollutant models (Model 1) with different exposure averaging periods.

All models were adjusted for baseline age, birth year, calendar date of interview, sex, race/ethnicity, marital status, number of children, educational attainment, ownership of the primary residence at baseline, total household wealth at baseline, time-varying urbanicity levels, time-varying NSES, and space with 10 df.

© 2024 Zhang B et al. *JAMA Network Open.*

Tables

eTable 1. Correlations among 10-year average air pollutants for respondents of the Health and Retirement Study (1998-2016).

	**Total $PM_{2.5}$**	**Agriculture**	**Road**	**NRTR**	**Energy_coal**	**Energy_other**	**Industry_coal**	**Industry_other**	**Wildfires**	**Dust**	**$PM_{10-2.5}$**	**NO_2**	**O_3**
**Total $PM_{2.5}$**	1	0.51	0.76	0.52	0.43	0.57	0.75	0.73	0.17	-0.01	0.02	0.62	-0.43
**Agriculture**		1	0.77	0.88	0.75	0.57	0.04	0.01	0.03	-0.30	-0.21	-0.04	-0.06
**Road**			1	0.80	0.76	0.65	0.41	0.32	-0.11	-0.10	-0.07	0.24	-0.19
**NRTR**				1	0.69	0.61	0.00	0.09	-0.08	-0.36	-0.29	0.01	-0.24
**Energy_coal**					1	0.58	0.03	-0.10	-0.20	-0.25	-0.26	-0.12	0.00
**Energy_other**						1	0.17	0.24	-0.26	-0.21	-0.14	0.20	-0.34
**Industry_coal**							1	0.84	0.09	0.26	0.33	0.67	-0.23
**Industry_other**								1	-0.05	0.09	0.12	0.79	-0.51
**Wildfires**									1	-0.05	0.10	0.06	0.04
**Dust**										1	0.66	0.24	0.18
**$PM_{10-2.5}$**											1	0.21	0.16
**NO_2**												1	-0.59
**O_3**													1

[a.] Abbreviations for the name of source: The sum of $PM_{2.5}$ form all other sources (Other); Road Traffic (Road); Non-Road Traffic (NRTR); Windblown Dust (Dust).

© 2024 Zhang B et al. *JAMA Network Open.*

eTable 2. Risk ratios for lost independence between 1998 and 2016 associated with an IQR increase in 10-year average of air pollutants in the Health and Retirement Study modified by baseline age.

	All (Model 1)	Baseline age < 75	Baseline age≥75	P
**Total $PM_{2.5}$**	1.05 (1.01, 1.10)	1.13 (1.08, 1.19)	1.00 (0.94, 1.05)	<0.001
**Agriculture**	1.02 (0.94, 1.11)	1.14 (1.06, 1.23)	1.06 (0.98, 1.15)	0.02
**Road Traffic**	1.09 (1.03, 1.16)	1.21 (1.13, 1.28)	1.04 (0.97, 1.11)	<0.001
**Non-road Traffic**	1.13 (1.03, 1.24)	1.28 (1.18, 1.38)	1.18 (1.09, 1.28)	0.01
**Energy coal**	1.01 (0.95, 1.06)	1.08 (1.02, 1.14)	1.01 (0.95, 1.06)	0.01
**Energy other**	1.05 (1.00, 1.11)	1.16 (1.10, 1.22)	1.01 (0.95, 1.07)	<0.001
**Industry Coal**	1.02 (0.99, 1.06)	1.04 (1.01, 1.08)	0.98 (0.95, 1.02)	0.00
**Industry Other**	1.02 (1.00, 1.04)	1.03 (1.01, 1.05)	0.99 (0.97, 1.02)	0.03
**Wildfires**	0.98 (0.96, 1.00)	1.00 (0.98, 1.02)	1.00 (0.98, 1.03)	0.68
**Windblown Dust**	1.00 (1.00, 1.01)	1.00 (1.00, 1.01)	1.00 (0.99, 1.01)	0.58
**$PM_{10-2.5}$**	1.02 (0.98, 1.05)	0.99 (0.96, 1.03)	0.98 (0.94, 1.02)	0.59
**NO_2**	1.05 (1.01, 1.08)	1.05 (1.01, 1.09)	0.95 (0.91, 0.99)	<0.001
**O_3**	0.94 (0.92, 0.97)	0.83 (0.80, 0.87)	0.85 (0.82, 0.89)	0.35

a. All models were adjusted for baseline age, birth year, calendar date of interview, sex, race/ethnicity, marital status, number of children, educational attainment, ownership of the primary residence at baseline, total household wealth at baseline, time-varying urbanicity levels, time-varying NSES, and space with 10 df.

© 2024 Zhang B et al. *JAMA Network Open.*

eTable 3. Risk ratios for lost independence between 1998 and 2016 associated with an IQR increase in 10-year average of air pollutants in the Health and Retirement Study modified by common chronic diseases.

	Prevalent CVD			Prevalent chronic lung diseases			Prevalent diabetes		
	**Without**	**With**	**P**	**Without**	**With**	**P**	**Without**	**With**	**P**
**Total $PM_{2.5}$**	1.01 (0.96, 1.07)	1.12 (1.06, 1.18)	0.00	1.05 (1.00, 1.10)	1.04 (0.96, 1.14)	0.96	1.03 (0.98, 1.08)	1.12 (1.05, 1.19)	0.01
**Agriculture**	0.99 (0.9, 1.09)	1.06 (0.96, 1.18)	0.07	1.02 (0.93, 1.12)	1.01 (0.88, 1.14)	0.80	1.01 (0.92, 1.11)	1.06 (0.95, 1.19)	0.28
**Road Traffic**	1.06 (0.99, 1.14)	1.13 (1.05, 1.22)	0.04	1.09 (1.02, 1.16)	1.07 (0.97, 1.18)	0.69	1.07 (1.00, 1.14)	1.17 (1.01, 1.27)	0.01
**Non-road Traffic**	1.10 (1.00, 1.21)	1.13 (1.03, 1.24)	0.46	1.13 (1.03, 1.24)	1.11 (0.99, 1.24)	0.66	1.11 (1.02, 1.21)	1.17 (1.05, 1.3)	0.20
**Energy coal**	0.99 (0.93, 1.05)	1.02 (0.96, 1.09)	0.25	1.01 (0.95, 1.07)	0.97 (0.89, 1.06)	0.29	1.00 (0.94, 1.06)	1.05 (0.98, 1.13)	0.09
**Energy other**	1.03 (0.98, 1.09)	1.08 (1.02, 1.15)	0.09	1.05 (0.99, 1.1)	1.07 (0.98, 1.17)	0.52	1.03 (0.98, 1.09)	1.11 (1.03, 1.19)	0.03
**Industry Coal**	1.01 (0.97, 1.04)	1.07 (1.02, 1.11)	0.00	1.02 (0.99, 1.05)	1.02 (0.95, 1.08)	0.93	1.02 (0.98, 1.06)	1.05 (1.00, 1.1)	0.21
**Industry Other**	1.01 (0.98, 1.03)	1.04 (1.01, 1.07)	0.03	1.02 (0.99, 1.04)	1.02 (0.97, 1.07)	0.99	1.01 (0.99, 1.04)	1.04 (1.01, 1.08)	0.15
**Wildfires**	0.97 (0.95, 1.00)	0.99 (0.96, 1.02)	0.14	0.98 (0.96, 1.00)	0.98 (0.94, 1.02)	0.96	0.98 (0.96, 1.00)	0.98 (0.94, 1.01)	0.68
**Windblown Dust**	1.00 (0.99, 1.01)	1.00 (1.00, 1.01)	0.35	1.00 (1.00, 1.01)	1.01 (1.00, 1.01)	0.19	1.00 (0.99, 1.01)	1.01 (1.00, 1.01)	0.12
**$PM_{10-2.5}$**	1.01 (0.96, 1.05)	1.01 (0.97, 1.06)	0.78	1.02 (0.98, 1.06)	1.01 (0.95, 1.07)	0.75	1.00 (0.96, 1.04)	1.04 (0.99, 1.09)	0.12
**NO_2**	1.03 (0.99, 1.07)	1.07 (1.00, 1.11)	0.10	1.04 (1.01, 1.08)	1.04 (0.98, 1.12)	0.93	1.03 (1.00, 1.07)	1.08 (1.02, 1.13)	0.12
**O_3**	0.94 (0.91, 0.98)	0.95 (0.92, 0.99)	0.68	0.94 (0.91, 0.98)	0.94 (0.88, 1.01)	0.98	0.96 (0.92, 0.99)	0.92 (0.87, 0.97)	0.16

a. All models were adjusted for baseline age, birth year, calendar date of interview, sex, race/ethnicity, marital status, number of children, educational attainment, ownership of the primary residence at baseline, total household wealth at baseline, time-varying urbanicity levels, time-varying NSES, and space with 10 df.

© 2024 Zhang B et al. *JAMA Network Open.*

eTable 4. Risk ratios for lost independence between 1998 and 2016 associated with an IQR increase in 10-year average of air pollutants in the Health and Retirement Study further adjusting for common chronic diseases.

	**Model 1**	**Model 1 +Prevalent CVD**	**Model 1 +Prevalent chronic lung diseases**	**Model 1 +Prevalent diabetes**
**Total $PM_{2.5}$**	1.05 (1.01, 1.10)	1.05 (1, 1.1)	1.05 (1.00, 1.10)	1.05 (1.01, 1.10)
**Agriculture**	1.02 (0.94, 1.11)	1.02 (0.93, 1.12)	1.02 (0.93, 1.11)	1.02 (0.94, 1.12)
**Road Traffic**	1.09 (1.03, 1.16)	1.09 (1.02, 1.16)	1.09 (1.02, 1.16)	1.09 (1.03, 1.17)
**Non-road Traffic**	1.13 (1.03, 1.24)	1.11 (1.02, 1.22)	1.13 (1.03, 1.23)	1.12 (1.03, 1.23)
**Energy coal**	1.01 (0.95, 1.06)	1.00 (0.95, 1.06)	1.00 (0.95, 1.06)	1.01 (0.95, 1.07)
**Energy other**	1.05 (1.00, 1.11)	1.05 (1.00, 1.11)	1.05 (1.00, 1.11)	1.05 (1.00, 1.11)
**Industry Coal**	1.02 (0.99, 1.06)	1.03 (0.99, 1.06)	1.02 (0.99, 1.05)	1.03 (0.99, 1.06)
**Industry Other**	1.02 (1.00, 1.04)	1.02 (1.00, 1.04)	1.02 (0.99, 1.04)	1.02 (1.00, 1.04)
**Wildfires**	0.98 (0.96, 1.00)	0.98 (0.96, 1,00)	0.98 (0.96, 1.00)	0.98 (0.96, 1.00)
**Windblown Dust**	1.00 (1.00, 1.01)	1,00 (1.00, 1.01)	1.00 (1.00, 1.01)	1.00 (1.00, 1.01)
**$PM_{10-2.5}$**	1.02 (0.98, 1.05)	1.01 (0.97, 1.05)	1.02 (0.98, 1.06)	1.01 (0.97, 1.05)
**NO_2**	1.05 (1.01, 1.08)	1.04 (1.01, 1.08)	1.04 (1.01, 1.08)	1.04 (1.01, 1.08)
**O_3**	0.94 (0.92, 0.97)	0.95 (0.92, 0.98)	0.94 (0.91, 0.97)	0.95 (0.92, 0.98)

a. Model 1 were adjusted for baseline age, birth year, calendar date of interview, sex, race/ethnicity, marital status, number of children, educational attainment, ownership of the primary residence at baseline, total household wealth at baseline, time-varying urbanicity levels, time-varying NSES, and space with 10 df.

© 2024 Zhang B et al. *JAMA Network Open.*

eTable 5. Hazard ratios for lost independence between 1998 and 2016 associated with an IQR increase in 10-year average of air pollutants in the Health and Retirement Study.

	Single-Pollutant Model (Model 1)	Model 1 + $PM_{2.5}$ (Model 2)	Model2+$PM_{10-2.5}$+O_3 (Model 3)
**Total $PM_{2.5}$**	1.04 (1.01, 1.08)		0.97 (0.91, 1.02)
**Agriculture**	0.99 (0.89, 1.10)	0.97 (0.85, 1.07)	0.98 (0.87, 1.09)
**Road Traffic**	1.08 (1.01, 1.15)	1.08 (0.96, 1.22)	1.08 (0.96, 1.23)
**Non-road Traffic**	1.08 (0.98, 1.20)	1.05 (0.93, 1.19)	1.03 (0.92, 1.16)
**Energy coal**	1.00 (0.94, 1.06)	0.98 (0.92, 1.05)	0.98 (0.92, 1.04)
**Energy other**	1.01 (0.95, 1.07)	0.98 (0.90, 1.06)	0.96 (0.89, 1.04)
**Industry Coal**	1.03 (1.00, 1.06)	1.00 (0.93, 1.08)	1.00 (0.92, 1.08)
**Industry Other**	1.02 (0.99, 1.04)	1.01 (0.97, 1.05)	0.99 (0.94, 1.03)
**Wildfires**	0.99 (0.96, 1.01)	0.99 (0.96, 1.01)	0.99 (0.96, 1.01)
**Windblown Dust**	1.00 (1.00, 1.01)	1.01 (1.00, 1.01)	1.00 (0.99, 1.01)
**$PM_{10-2.5}$**	1.03 (0.97, 1.08)	1.02 (0.97, 1.07)	1.02 (0.97, 1.07)
**NO_2**	1.06 (1.02, 1.09)	1.07 (1.01, 1.13)	1.02 (0.95, 1.10)
**O_3**	0.94 (0.91, 0.97)	0.93 (0.89, 0.98)	0.94 (0.89, 1.00)

a. All models were stratified by 2-year age at baseline, 2-year birth cohort, and adjusted for sex, race/ethnicity, marital status, number of children, educational attainment, ownership of the primary residence at baseline, total household wealth at baseline, time-varying urbanicity levels, time-varying NSES and space with 10 df.

© 2024 Zhang B et al. *JAMA Network Open.*

eTable 6. Risk ratios for lost independence between 1998 and 2016 associated with an IQR increase in 10-year average of air pollutants in the Health and Retirement Study modified when restricting to those not socially isolated.

	All (Model 1)	Married/Partnered Or Children≥ 1	Married/Partnered	Children≥ 1
**Total $PM_{2.5}$**	1.05 (1.01, 1.10)	1.06 (1.01, 1.11)	1.03 (0.98, 1.09)	1.05 (1.01, 1.10)
**Agriculture**	1.02 (0.94, 1.11)	1.04 (0.96, 1.14)	1.05 (0.95, 1.16)	1.04 (0.96, 1.14)
**Road Traffic**	1.09 (1.03, 1.16)	1.11 (1.04, 1.18)	1.08 (1.00, 1.16)	1.10 (1.03, 1.17)
**Non-road Traffic**	1.13 (1.03, 1.24)	1.17 (1.07, 1.28)	1.17 (1.05, 1.30)	1.16 (1.06, 1.27)
**Energy coal**	1.01 (0.95, 1.06)	1.01 (0.95, 1.07)	0.99 (0.92, 1.05)	1.01 (0.95, 1.07)
**Energy other**	1.05 (1.00, 1.11)	1.07 (1.02, 1.12)	1.06 (0.99, 1.12)	1.07 (1.02, 1.13)
**Industry Coal**	1.02 (0.99, 1.06)	1.03 (1.00, 1.06)	1.01 (0.97, 1.05)	1.02 (0.99, 1.06)
**Industry Other**	1.02 (1.00, 1.04)	1.02 (1.00, 1.05)	1.01 (0.99, 1.04)	1.02 (1.00, 1.04)
**Wildfires**	0.98 (0.96, 1.00)	0.98 (0.96, 1.00)	0.98 (0.95, 1.00)	0.98 (0.96, 1.00)
**Windblown Dust**	1.00 (1.00, 1.01)	1.00 (1.00, 1.01)	1.00 (0.99, 1.00)	1.00 (1.00, 1.01)
**$PM_{10-2.5}$**	1.02 (0.98, 1.05)	1.02 (0.98, 1.05)	1.00 (0.96, 1.04)	1.02 (0.98, 1.06)
**NO_2**	1.05 (1.01, 1.08)	1.05 (1.01, 1.08)	1.04 (1.00, 1.08)	1.04 (1.01, 1.08)
**O_3**	0.94 (0.92, 0.97)	0.94 (0.91, 0.97)	0.96 (0.92, 0.99)	0.94 (0.91, 0.97)

a. All models were adjusted for baseline age, birth year, calendar date of interview, sex, race/ethnicity, marital status, number of children, educational attainment, ownership of the primary residence at baseline, total household wealth at baseline, time-varying urbanicity levels, time-varying NSES, and space with 10 df.

© 2024 Zhang B et al. *JAMA Network Open.*

eTable 7. The risk of loss of independence per quartile increases in 10-year average of O_3 in the Health and Retirement.

	Single-Pollutant Model (Model 1)	Model 1 + $PM_{2.5}$ (Model 2)	Model2+$PM_{10-2.5}$+O_3 (Model 3)
Primary Results	0.94 (0.92, 0.97)	0.94 (0.91, 0.98)	0.95 (0.91, 0.99)
+max NDVI with 250m buffer	0.95 (0.91, 0.99)	0.95 (0.91, 0.99)	0.94 (0.89, 1.00)
+max NDVI with 1000m buffer	0.94 (0.91, 0.98)	0.94 (0.90, 0.99)	0.94 (0.89, 0.99)
+annual mean temperature	0.95 (0.92, 0.99)	0.95 (0.91, 0.99)	0.94 (0.89, 1.00)

a. Model 1 were adjusted for baseline age, birth year, calendar date of interview, sex, race/ethnicity, marital status, number of children, educational attainment, ownership of the primary residence at baseline, total household wealth at baseline, time-varying urbanicity levels, time-varying NSES, and space with 10 df.

© 2024 Zhang B et al. *JAMA Network Open.*

eTable 8. Risk ratios for lost independence in the Health and Retirement Study associated with an IQR increase in average concentrations of $PM_{2.5}$ from wildfires estimated with time-fixed emission data (primary) and wildfires estimated with time-varying emission data (2006 to 2016) with different average periods and baseline.

	**Baseline**	**Wildfires $PM_{2.5}$ (Fixed Estimates)**	**Wildfires $PM_{2.5}$ (Time-Varying Estimates)**
**10-year average**	2014	1.05 (0.99, 1.10)	1.02 (0.95, 1.10)
**5-year average**	2010	1.02 (0.98, 1.06)	1.01 (0.95, 1.06)
**1-year average**	2006	1.00 (0.98, 1.03)	1.0 0.94, 1.06)

a. All models were adjusted for baseline age, birth year, calendar date of interview, sex, race/ethnicity, marital status, number of children, educational attainment, ownership of the primary residence at baseline, total household wealth at baseline, time-varying urbanicity levels, time-varying NSES, and space with 10 df..

We evaluated the association of wildfire-related $PM_{2.5}$ with lost independence using time-varying fires data from another published source[3]. Since this time-varying fires data was only available from 2006 to 2018, we set 2014 as the baseline since this would allow us to calculate a 10-year average exposure when allowing for <75% non-missing data. To facilitate the comparison between the results using this time-varying fires data and our primary exposure estimates using fixed fractional contribution, we also limited the analyses with our primary fire-related $PM_{2.5}$ estimates to the same baseline. We also make similar comparisons using 5-year and 1-year exposure periods with 2010 and 2006 as the earliest baseline respectively.

© 2024 Zhang B et al. *JAMA Network Open.*

eTable 9. Risk ratios for moving to nursing homes between 1998 and 2016 associated with an IQR increase in 10-year average of air pollutants in the Health and Retirement Study.

	All (Main Outcome)	Moving to NH
**Total $PM_{2.5}$**	1.05 (1.01, 1.10)	1.13 (1.00, 1.28)
**Agriculture**	1.02 (0.94, 1.11)	1.02 (0.93, 1.12)
**Road Traffic**	1.09 (1.03, 1.16)	1.13 (1.03, 1.23)
**Non-road Traffic**	1.13 (1.03, 1.24)	1.09 (1.02, 1.17)
**Energy coal**	1.01 (0.95, 1.06)	1.01 (0.95, 1.06)
**Energy other**	1.05 (1.00, 1.11)	1.05 (0.95, 1.06)
**Industry Coal**	1.02 (0.99, 1.06)	1.05 (1.00, 1.11)
**Industry Other**	1.02 (1.00, 1.04)	1.02 (0.99, 1.06)
**Wildfires**	0.98 (0.96, 1.00)	0.98 (0.96, 1.00)
**Windblown Dust**	1.00 (1.00, 1.01)	1.00 (1.00, 1.01)
**$PM_{10-2.5}$**	1.02 (0.98, 1.05)	1.02 (0.98, 1.05)
**NO_2**	1.05 (1.01, 1.08)	1.05 (1.01, 1.08)
**O_3**	0.94 (0.92, 0.97)	0.94 (0.91, 0.98)

© 2024 Zhang B et al. *JAMA Network Open.*

Reference

1. McDuffie EE, Martin RV, Spadaro JV, et al. Source sector and fuel contributions to ambient PM2. 5 and attributable mortality across multiple spatial scales. *Nature Communications*. 2021;12(1):1-12.
2. Hurd MD, Martorell P, Delavande A, Mullen KJ, Langa KM. Monetary costs of dementia in the United States. *New England Journal of Medicine*. 2013;368(14):1326-1334.
3. O'Dell K, Ford B, Fischer EV, Pierce JR. Contribution of Wildland-Fire Smoke to US PM2.5 and Its Influence on Recent Trends. *Environmental Science & Technology*. 2019/02/19 2019;53(4):1797-1804. doi:10.1021/acs.est.8b05430

© 2024 Zhang B et al. *JAMA Network Open.*
