# Supplementary material for: Source-Specific Air Pollution and Loss of Independence in Older Adults Across the US
Source: JAMA Netw Open. 2024 Jun 28;7(6):e2418460. doi: 10.1001/jamanetworkopen.2024.18460 (PMC11214115; doi:10.1001/jamanetworkopen.2024.18460)
Supplement: Supplement 2. — Data Sharing Statement [file jamanetwopen-e2418460-s002.pdf]

Data Sharing Statement

Zhang. Source-Specific Air Pollution and Loss of Independence in Older Adults Across the US. *JAMA Netw Open*. Published June 28, 2024. doi:10.1001/jamanetworkopen.2024.18460

Data
Data available: No
